# Multifaceted roles of WRKY transcription factors in abiotic stress and flavonoid biosynthesis

**DOI:** 10.3389/fpls.2023.1303667

**Published:** 2023-12-15

**Authors:** Jinnan Zhang, Haiqing Zhao, Lu Chen, Jiacheng Lin, Zhile Wang, Jiaqi Pan, Fan Yang, Xiaoli Ni, Yiang Wang, Yuhua Wang, Rui Li, Erxu Pi, Shang Wang

**Affiliations:** College of Life and Environmental Sciences, Hangzhou Normal University, Hangzhou, China

**Keywords:** abiotic stresses, *cis*-elements, flavonoids biosynthesis, transcription factor, WRKY family

## Abstract

Increasing biotic and abiotic stresses are seriously impeding the growth and yield of staple crops and threatening global food security. As one of the largest classes of regulators in vascular plants, WRKY transcription factors play critical roles governing flavonoid biosynthesis during stress responses. By binding major W-box *cis*-elements (TGACCA/T) in target promoters, WRKYs modulate diverse signaling pathways. In this review, we optimized existing WRKY phylogenetic trees by incorporating additional plant species with WRKY proteins implicated in stress tolerance and flavonoid regulation. Based on the improved frameworks and documented results, we aim to deduce unifying themes of distinct WRKY subfamilies governing specific stress responses and flavonoid metabolism. These analyses will generate experimentally testable hypotheses regarding the putative functions of uncharacterized WRKY homologs in tuning flavonoid accumulation to enhance stress resilience.

## 1 Introduction

Escalating stresses seriously impede the production of important crops, threatening global food security (Zhang et al., 2022a). Plants have evolved intricate regulatory networks. The WRKY family, as an important member of these networks, belongs to the functionally diversified transcription factor families. WRKYs are critical in governing plant diverse stress responses (Jiang and Deyholos, 2009; Li et al., 2009; Pandey and Somssich, 2009; Hu et al., 2013; Li et al., 2013; Yokotani et al., 2013; Bakshi and Oelmüller, 2014; Dai et al., 2016; Ahammed et al., 2020b; Wu et al., 2021; Ma et al., 2021c; Lin et al., 2022; Ma et al., 2023).

In sweet potato (*Ipomoea batatas*), after the inaugural gene was found (Ishiguro and Nakamura, 1994), WRKYs have been discovered extensively in terrestrial plants, primitive protozoans and slime molds, affirming the ancient evolutionary origin (Zhang and Wang, 2005; Pan et al., 2009). Highly conserved WRKY structural domains paralleling the zinc finger (ZF) motif is an essential feature of this protein family. The WRKY domain directly bind to the major *cis*-element, W-box (TGACCA/T) (Rushton et al., 2010; Chen et al., 2019). Although slight variations in some WRKYs, the conserved motif (WRKYGQK) forms the ZF to confer structural stability (Yamasaki et al., 2005; Cheng et al., 2021).

Phylogenetically, WRKYs are classified into three distinct groups depended on the number of WRKY domains together with the type of ZFs. Two WRKY domains and a C2H2 (CX4-5CX22-23HXH) ZF are present in Group I, whereas only one WRKY domain combined with C2H2 or altered C2HC (CX7CX23HXC) ZF appears in Groups II and III (Eulgem et al., 2000; Li et al., 2010; Chen et al., 2020). According to the variation of ZF, Group II can be subclassified into subgroups IIa ~ IIe (Zhang and Wang, 2005). The presence of Group I WRKYs in primitive organisms suggests that they are the ancestors of other groups (Wei et al., 2018). WRKY domains are conserved, but different WRKY groups have evolved specialized functions (He et al., 2016; Wang et al., 2022).

The importance of WRKYs in modulating plant immunological responses against pathogen invasion, including effector-triggered immunity (ETI) and pathogen-associated molecular pattern (PAMP)-triggered immunity (PTI), have been proven (Chen et al., 2013; Chi et al., 2013; Dang et al., 2013; Birkenbihl et al., 2017; Ma et al., 2021b; Wang et al., 2023b; Wang et al., 2023d; Xiao et al., 2023). Numerous WRKY genes spanning all phylogenetic groups are induced following pathogen infection or elicitor treatments across plant species, underscoring the broad defensive role (Chen et al., 2013; Dang et al., 2013; Birkenbihl et al., 2017; Wang et al., 2023b; Wang et al., 2023d; Xiao et al., 2023). Gain- and loss-of-function analyses of WRKYs have demonstrated both positive and negative regulatory functions in immune signaling (Shen et al., 2023). Additionally, WRKYs govern hormone signaling in plant (Shang et al., 2010; Chen et al., 2013; Li et al., 2013; Ding et al., 2015; Zhang et al., 2015; Chen et al., 2017; Wang et al., 2023c), regulate secondary metabolism (Suttipanta et al., 2011; Wang et al., 2023d), and dominate stress responses (Jiang and Deyholos, 2009; Li et al., 2020c; Ma et al., 2023).

Although the functional roles of plant WRKY transcription factors in stress responses are well-documented, the relationship between sequence divergences across distinct WRKY domains and their varying biological activities is still unclear. In order to fully understand the functional specificity encoded within different WRKY phylogenetic clades, further investigation is necessary Furthermore, as critical homeostatic regulators of ROS, numerous flavonoid biosynthetic genes harbor abundant W-box *cis*-elements within their promoters (Liu et al., 2019a; Su et al., 2022a). Growing evidence suggests that specific WRKY proteins play pivotal roles governing flavonoid metabolism to enhance plant stress adaptation (An et al., 2019; Wang et al., 2022). However, the specific contributions of individual members of the WRKY subfamily in directing flavonoid accumulation are not well understood. In this review, we will refine WRKY phylogenetic frameworks by incorporating additional plant species with WRKY genes that have been implicated in stress tolerance and flavonoid regulation. Through these analyses, we aim to elucidate the roles of specific WRKY clades in modulating flavonoid biosynthesis under defined abiotic stresses.

## 2 Phylogenetic analysis of WRKY proteins

The WRKYs sequences of Arabidopsis *(Arabidopsis thaliana)*, poplar *(Populus trichocarpa)*, maize *(Zea mays)*, rice *(Oryza sativa)*, and soybean *(Glycine max)* were downloaded from PlantTFDB (http://planttfdb.gao-lab.org/index.php). Protein sequences of WRKY with certain functions mentioned in this review were sourced from NCBI (https://www.ncbi.nlm.nih.gov). To distinguish the presence and number of structural WRKY domains, the sequences of WRKYs were examined by NCBI CD-search (https://www.ncbi.nlm.nih.gov/Structure/bwrpsb/bwrpsb.cgi) and SMART (http://smart.embl.de/). Proteins containing the WRKY domain can be used for phylogenetic analysis. By using bootstrap (1, 000 replicates), building a phylogenetic tree requires the use of NJ method by MEGA 7.0 software. Finally, the phylogenetic tree was visualized and embellished using Interactive Tree of Life (iTOL, https://itol.embl.de/) (Letunic and Bork, 2007; Nan et al., 2020).

**Figure 1:**
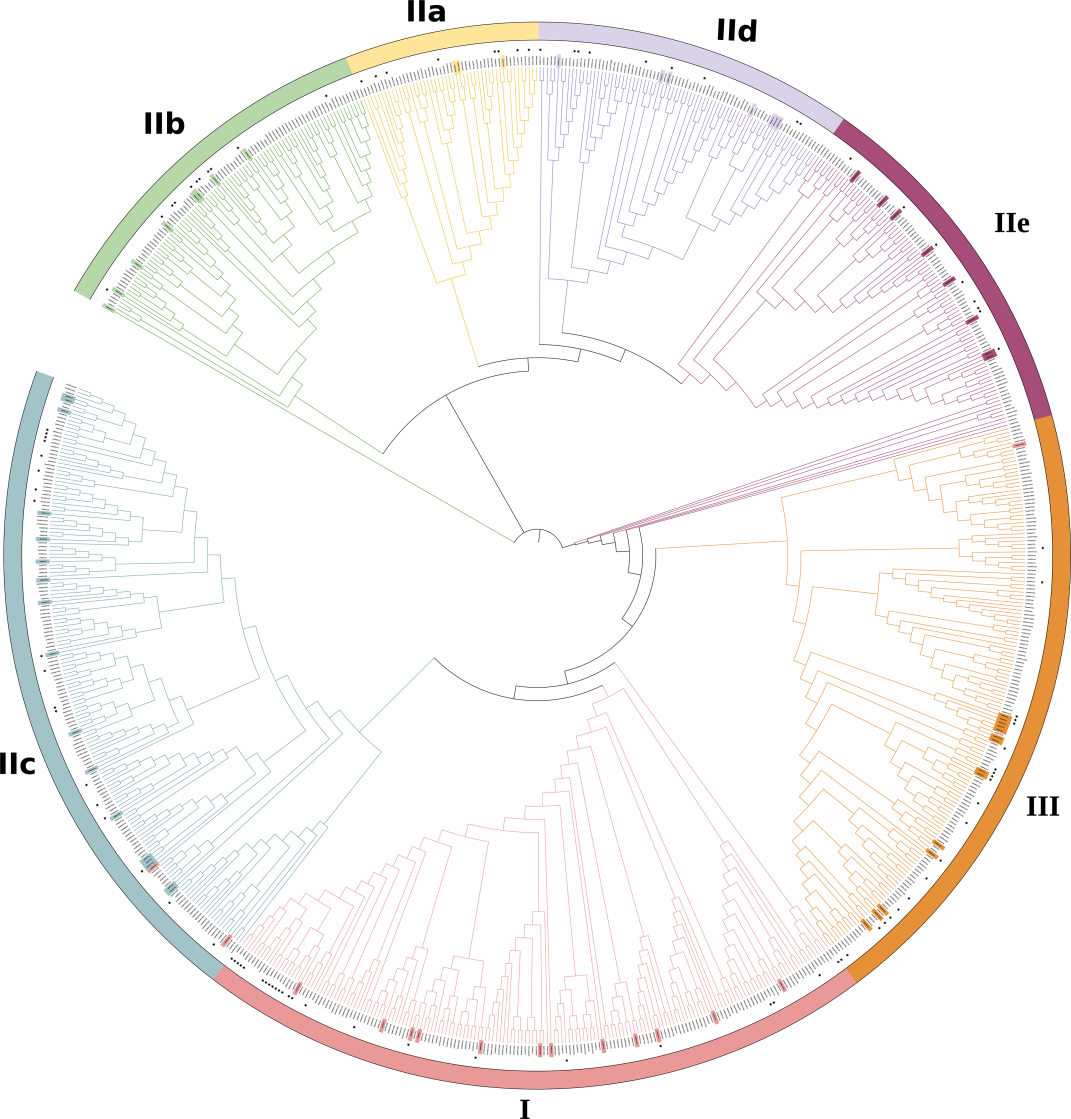
Phylogenetic analysis of identified WRKY proteins in *Arabidopsis thaliana, Glycine max, Oryza sativa, Populus trichocarpa* and *Zea mays*. A phylogenetic tree was established using the MEGA 7.0 program and the neighborhood joining method, drawing on WRKY domain sequences. Different colors have been utilized to distinguish between various groups or subgroups. WRKY protein sequences were obtained from NCBI (https://www.ncbi.nlm.nih.gov) database. The 72 proteins highlighted in the inner circle originate from Arabidopsis thaliana (**Supplementary Table S1**), while WRKY genes from Groups I to III are color-coded in **Supplementary Table S2**. The functional categories of WRKY, annotated in this review, are identified by black asterisks in **Supplementary Table S3**. **Supplementary Table S4** provides a comprehensive list of the protein IDs and full sequences for all analyzed WRKYs (Jeyasri et al., 2021; He et al., 2012; Zhang et al., 2017; Yu et al., 2016).

The phylogenetic analysis showed that the 816 WRKYs were segregated into three canonical groups, designated as Roman numerals (I, II, III), with seven distinct groups and represented by colored outer circles (**Figure 1**), consistent with the phylogenetic system defined by Zhang and Wang (2005). Further examination reveals that Group II can be categorized into five unique subdivisions: IIa, IIb, IIc, IId, and IIe (**Figure 1**). Subgroup I, with 172 members, is the most numerous of the 7 subgroups. Whereas groups IIa contained only 49 members. The further division of Group II into 5 subgroups is due to specific sequence variations in the ZF motifs (Xie et al., 2005).

When compared to Zhang and Wang’s system (Zhang and Wang (2005)), three WRKY proteins of the present phylogenetic analysis had distinct classes. For example, AtWRKY10, AtWRKY19, AtWRKY45 from subgroup 1 are reassigned to II c, II c, and III, respectively.

## 3 WRKYs bind to specific *cis*-acting regions of target genes

A platform for systematic investigations of WRKY family is provided by the expanding genome sequencing data from many plant species. The identification of genes crucial for specific processes has been made possible by genome sequences, which have enabled transcriptome profiling of some families (such as WRKY) during particular situations (Arndt et al., 2022; Shende et al., 2022; Zhang et al., 2022e). Furthermore, the genome-wide mapping of WRKY binding sites and target genes has been made possible by high-throughput sequencing after chromatin immunoprecipitation (ChIP-seq) (Zhou et al., 2021; Xu et al., 2022). These cutting-edge approaches have profoundly expanded the understanding of WRKY gene functions and regulatory networks.

The C-terminus contains the ZF structure C2H2 or C2HC, while the WRKYGQK motif, which forms the core region, is present at the N-terminus (Yamasaki et al., 2005; Chen et al., 2010; Song et al., 2018; Gu et al., 2019; Wang et al., 2021a). Some WRKY proteins also contain WRRY, WSKY, WKRY, WVKY, or WKKY motifs in place of the WRKY domain (Xie et al., 2005; Duan et al., 2007; Li et al., 2020b). The binding of DNA by WRKY proteins necessitates the ZF motif (Phukan et al., 2017). Replacing the zinc ion in WRKY domains with the metal chelator 1,10-phenanthroline abolishes DNA binding, indicating that WRKY proteins possess ZFs structures. The WRKY domain is composed of four β-sheets, a zinc-binding pocket, and coordination with cysteine or histidine residues make up the WRKY domain. The C-terminal of the WRKY domain stabilizes the sequence-specific interaction between the projecting N-terminal WRKYGQK sequence and the 6 bp DNA groove. Therefore, WRKY proteins may attach to the W-box clusters, primarily the TTGACC/T motif, in promoters of target genes. This enables them to regulate the dynamic network of signals and responses (Yamasaki et al., 2005; Li et al., 2020b).

The core TGAC sequence is highly conserved and principally responsible for binding by WRKY proteins (Mare et al., 2004; Ciolkowski et al., 2008; Rahman et al., 2021). In contrast, variations in the number, sequence, and nucleotide composition of the flanking bases in W-boxes of target genes contribute to the binding specificities of different WRKYs (Cheng et al., 2019).

Indeed, most target genes of WRKY were discovered to contain W-box *cis*-elements in the promoters, as identified through various approaches. Chromatin immunoprecipitation analyses revealed that Parsley (*Petroselinum crispum*) WRKY1 binds to W-boxes in the promoters of *PcWRKY3* and *Pathogenesis-Related1-1* (*PcPR1-1*) (Turck et al., 2004), and pepper (*Capsicum annuum*) WRKY6 was able to bind to the W-boxes of the *CaWRKY40*, as well as defense genes *Capsicum annuum* defensin 1 (*CaDEF1*), EXTRACELLULAR PEROXIDASE 2 *(CaPO2)*, and small heat shock protein 24 (*CaHSP24)* (Hussain et al., 2018). Furthermore, pull-down assays was used to identify candidate W-box containing genes (*AtWRKY58, AtWRKY13, AtWRKY6*) of AtWRKY53 (Miao et al., 2004). Electrophoretic mobility shift assay was applied to uncover putative W-box targets of WRKY38 in barley (*Hordeum vulgare*) (Mare et al., 2004), WRKY26 (Li et al., 2011), WRKY11 (Ali et al., 2018), WRKY53 (Sun and Yu, 2015) in Arabidopsis, and WRKY42 (Su et al., 2015), WRKY1, WRKY2, and WRKY4 in tobacco (*Nicotiana tabacum*) (Yamamoto et al., 2004), as well as WRKY71 in rice (Liu et al., 2007).

**Figure 2:**
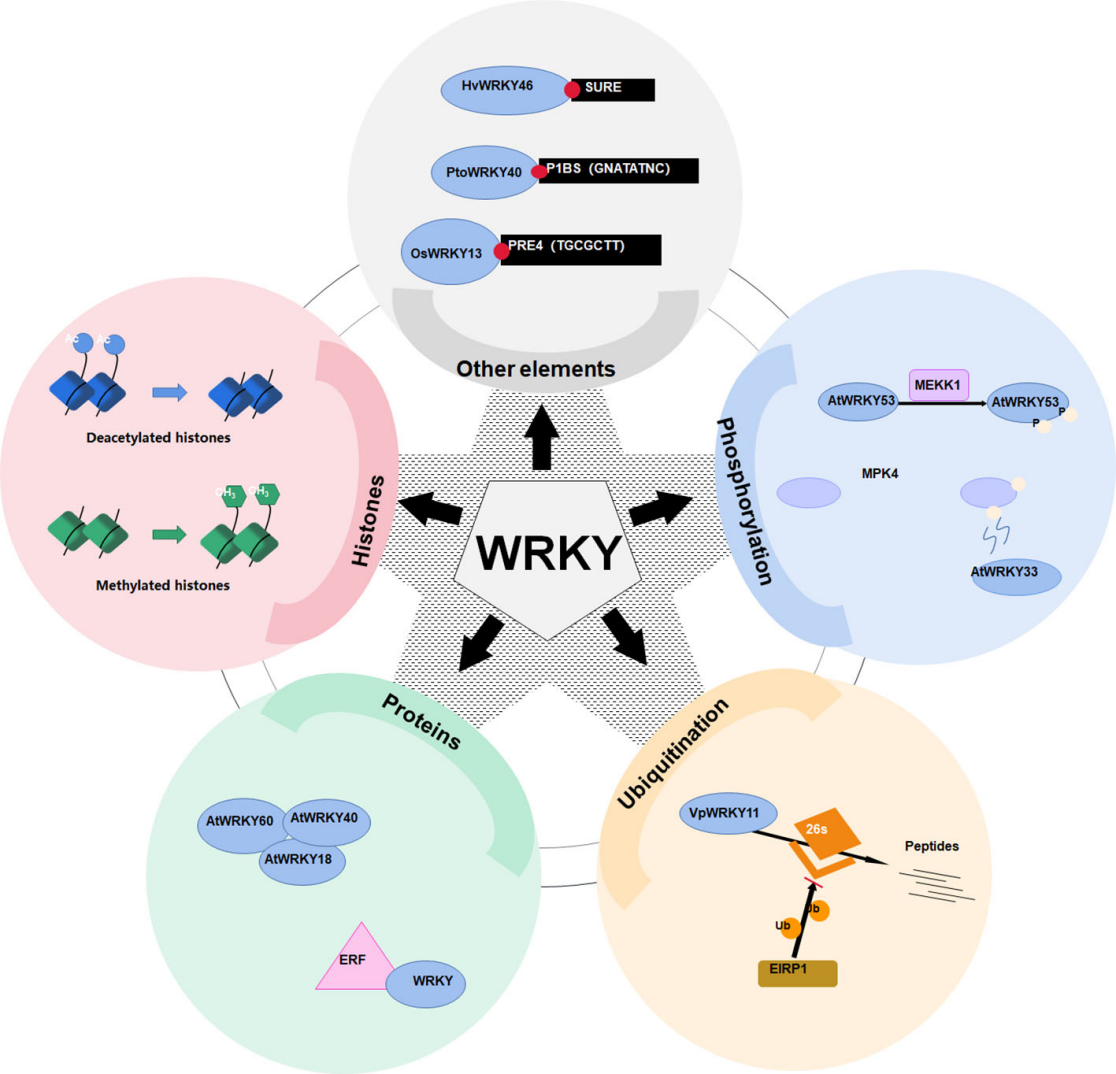
Various mechanisms of WRKY transcriptional activation regulation. WRKY transcription factors are regulated via a plethora of mechanisms. These range from binding to an assortment of *cis*-regulatory elements such as SURE, P1BS, and PRE4, to post-translational modifications including ubiquitination, methylation, and phosphorylation, which either activate them or mark them for degradation. Regulation may also involve altered chromatin conformations mediated by changes in histone acetylation or methylation, as well as modulation via interactions with other nuclear proteins like WRKY and ERF.

Except W-box, WRKY proteins could also recognize several other *cis*-elements (**Figure 2**). Rice OsWRKY13 binds the pathogen-responsive *cis*-element 4 (PRE4) (TACTGCGCTTAGT) (Xiao et al., 2013), while barley SUSIBA2/HvWRKY46 recognizes the sugar responsive element in the iso1 promoter (Sun et al. (2003). Moreover, PtoWRKY40 combines with the PHR1-binding site (P1BS) element (GNATATNC) (Zhou et al., 2008; Sun et al., 2016). The AtWRKY70 bound the WT-box sequence (YGACTTTT) of the Pep25-responsive gene expression in parsley protoplasts (Machens et al., 2014). In addition, the heat-inducible OsWRKY11 was found to attach to the promoter of heat shock elements (nGAAnnTTCnnGAAn), leading to enhanced thermotolerance of transgenic rice seedlings (Lee et al., 2018).

In essence, WRKY takes part in a variety of regulatory processes by regulating the expression of various *cis*-elements of target genes (**Table 1**). Current studies on the *cis*-elements of WRKY has primarily focused on six species, including *Oryza sativa*, *Arabidopsis thaliana*, *Petroselinum crispum*, *Hordeum vulgare*, *Nicotiana tabacum* and *Capsicum annuum*. It appears that members of WRKY subgroup 2e have a preference for the PRE4 element. WRKYs in subgroup 1 typically bind to the sulfur-responsive element (SURE), while the those in subgroup 3 interact with both the WT-box and the traditional W-box.

**Table 1:** Mechanism of WRKY on transcriptional regulation of target genes.

cis-acting element	Plant species	Nomenclature	Target gene	Regulation of target gene	Regulation of biological response	Subgroup	Effect	References
W-box (TTGACC/T)	*Oryza sativa*	OsWRKY11	CHT2, RAB21, PR10, Bet v1, HSP101	+	+	2c	play a pivotal role in the response to such stress. Participate in pathogens, drought, and heat tolerance	(Lee et al., 2018)
W-box (TTGACC/T)	*Arabidopsis thaliana*	AtWRKY42	PHT1, PHO1	+,-	–	2b	Regulating Phosphate Translocation and Acquisition in Arabidopsis	(Su et al., 2015)
W-box (TTGACC/T)	*Arabidopsis thaliana*	AtWRKY53	CAT1, CAT2, CAT3, QQS	+	–	3	negatively regulates drought tolerance by mediating stomatal movement	(Sun and Yu, 2015)
W-box (TTGACC/T)/W_BC_	*Petroselinum crispum*	PcWRKY1	PcPR1-1, PcWRKY3	–	/	1	Regulate plant responses to biotic stress and during senescence.	(Turck et al., 2004)
W-box (TTGACC/T)	*Prunus cerasus x Prunus canescens*	PcWRKY3	PcPR1-1, PcWRKY1	–	/	1	Regulate plant responses to biotic stress and during senescence.	(Turck et al., 2004)
W-box (TTGACC/T)	*Hordeum vulgare*	HvWRKY38	/	/	/	2a	Participate in cold and drought response	(Mare et al., 2004)
W-box (CTGACC/T)	*Nicotiana tabacum*	NtWRKY1	CHN48	+	/	1	Participate in elicitor-responsive transcription of defense genes in tobacco.	(Yamamoto et al., 2004)
W-box (CTGACC/T)	*Nicotiana tabacum*	NtWRKY2	CHN48	+	/	1	Participate in elicitor-responsive transcription of defense genes in tobacco.	(Yamamoto et al., 2004)
W-box (CTGACC/T)	*Nicotiana tabacum*	NtWRKY4	CHN48	+	/	3	Participate in elicitor-responsive transcription of defense genes in tobacco.	(Yamamoto et al., 2004)
CpNpG/W-box (TTGACC/T)	*Oryza sativa*	OsWRKY71	Amy32b	–	–	2a	participate in GA induction	(Zhang et al., 2004)
W-box (TTGACC/T)	*Oryza sativa*	OsWRKY45	DPF	+	+	3	Participate in abiotic stresses such as low temperature	(Cheng et al., 2019)
W-box (TTGACC/T)/SURE(TAAAGATTACTAATAGGAA)	*Hordeum vulgare*	HvWRKY46	/	/	/	1	/	(Pandey et al., 2018)
W-box (TTGACC/T)	*Capsicum annuum*	CaWRKY6	CaDEF1, CaPO2, CaHSP24	–	+	2b	Regulator in Pepper Response to *Ralstonia Solanacearum*	(Hussain et al., 2018)
W-box (TTGACC/T)	*Capsicum annuum*	CaWRKY40	CaDEF1, CaPO2, CaHSP24	–	+	2a	Regulator in Pepper Response to Ralstonia Solanacearum	(Hussain et al., 2018)
W-box (TTGACC/T)	*Arabidopsis thaliana*	AtWRKY6	PR1, SIRK	+	+	2b	Participate in the senescence- and defense-associated	(Robatzek and Somssich, 2002)
W-box (TTGACC/T)	*Arabidopsis thaliana*	AtWRKY11	/	/	+	2d	Participate in abiotic stress tolerance and regulation of plant defense responses.	(Ali et al., 2018)
W-box (TTGACC/T)	*Arabidopsis thaliana*	AtWRKY26	Hsp101, Hsp70, HsfA2, HsfB1, MBF1c, APX1, and Zat10	/	+	1	Participate in heat stress	(Li et al., 2011)
W-box (TTGACC/T)	*Arabidopsis thaliana*	AtWRKY38	PR1	–	–	3	Participate in Basal Defense	(Kim et al., 2008)
W-box (TTGACC/T)	*Arabidopsis thaliana*	AtWRKY43	/	/	/	2c	/	(Ciolkowski et al., 2008)
W-box (TTGACC/T)/PRE4(TACTGCGCTTAGT)	*Oryza sativa*	OsWRKY13	SNAC1	–	–	2e	Participate in the abiotic stress	(Xiao et al., 2013)
W-box (TTGACC/T)/WT-box(YGACTTTT)	*Arabidopsis thaliana*	AtWRKY70	/	/	–	3	Regulate leaf senescence	(Machens et al., 2014)
W-box (TTGACT/C)/P1BS(GNATATNC)	Populus tomentos a Carr.	PtoWRKY40	PtoPHT1s	–	–	2a	Participate in phosphorus deficiency response	(Chen et al., 2022)
W-box (TTGACC/T)	*Arabidopsis thaliana*	AtWRKY4	/	/	/	1	/	(Yamasaki et al., 2012)

Amy, amylases; APX, ascorbate peroxidases; Bet v, Betula verrucosa; CAT, catalase; CHN, class I chitinase; Cht, chitinase; DEF, defensin; DPF, diterpenoid phytoalexin factor; Hsf, heat stress transcription factor; HSP, heat shock protein; MBF1, multiprotein bridging factor; PHO1, a protein involved in loading inorganic phosphate (Pi) into the xylem of roots; PHT, phosphate transporter; PO, EXTRACELLULAR PEROXIDASE; PR, pathogenesis-related; QQS, Qua-Quine Starch; RAB, RESPONSIVE TO ABA; SIRK, A SENESCENCE-INDUCED RECEPTOR-LIKE KINASE; SNAC, tress-responsive NAC; Zat, zinc finger transcription factor. The ‘+’ represents positive regulation, the ‘-’ represents negative regulation, and the ‘/’ represents unknown.

## 4 Regulatory mechanism of WRKYs involved in their transactivation capabilities

Extensive researches unveiled that plant WRKYs were crucial orchestrators involved in two branches of plant innate immunity (PII) (Jones and Dangl, 2006).

Upon perception of PAMPs, the mitogen-activated protein kinase (MAPK) cascade is activated, leading to upregulation of AtWRKY22 and AtWRKY29, which are two positive regulators from 2e subgroup (Asai et al., 2002; Hsu et al., 2013). Certain WRKYs also fulfill indispensable regulatory roles in ETI (Adachi et al., 2015). A plethora of WRKY genes are auto-regulated or cross-regulated through W-box elements embedded in the promoters (Robatzek and Somssich, 2002; Turck et al., 2004; Besseau et al., 2012; Yang et al., 2012; Adachi et al., 2015). For instance, CaWRKY6 activates *CaWRKY40* to elevate pepper tolerance to heat and high humidity (Cai et al., 2015).

Post-translational modifications, especially phosphorylation, constitute key mechanisms orchestrating the transactivation capability of WRKYs. MAPKs phosphorylate WRKY proteins, either the DNA binding affinity is directly or indirectly altered, or the transcriptional activity is altered (Miao et al., 2007; Adachi et al., 2015). Prolonged MAPK activation increases the proportion of phosphorylated WRKYs (**Figure 2**), amplifying downstream signaling (Ishihama et al., 2011). For example, a MAPK kinase kinase 1 (MEKK1) phosphorylates AtWRKY53 to increase its DNA binding activity (Miao et al., 2007). In addition, MAPK 4 (MPK4) phosphorylates MKS1 (MAP kinase substrate 1) to liberate AtWRKY33 from the MKS1-WRKY33 complex, so as to targets the promoter of *PHYTOALEXIN DEFICIENT3* (*PAD3*) for the synthesis of antimicrobial camalexin (Qiu et al., 2008).

Proteasome-mediated degradation also governs WRKY levels (**Figure 2**). The E3 ubiquitin protein ligase 5 (UPL5) polyubiquitinates AtWRKY53, triggering its degradation (Adachi et al., 2015). Through the 26S proteasome, the E3 ligase Erysiphe necator-induced RING finger protein 1 (EIRP1) mediates the degradation of VpWRKY11 (Yu et al., 2013).

WRKY can fine-tune their transactivation capabilities by formation of polymers. WRKY-WRKY interactions are widespread (Xie et al., 2006; Chen et al., 2009). For example, WRKY proteins AtWRKY18, AtWRKY40 and AtWRKY60 interact both physically and functionally (**Figure 2**) (Xu et al., 2006; Chen et al., 2010). In addition, WRKY also interact with ethylene responsive factor (ERF) (Wang et al., 2023a) or VQ motif-containing proteins to regulate their activities (Lei et al., 2017).

In addition, histone modifications can also impact activities of WRKYs (**Figure 2**). For instance, the histone deacetylase 19 (HDA19) represses AtWRKY38 and AtWRKY62 (Kim et al., 2008). Additionally, the histone methyltransferase suppressor of variegation 3-9-homologous (SUVH2) epigenetically regulates AtWRKY53 (Ay et al., 2009).

In conclusion, WRKYs emerged as pivotal regulators in regulating plant responses to the environment. The diverse functions are fine-tuned through intricate interaction networks and post-translational modifications. Further elucidating these regulatory mechanisms will provide valuable insights into optimally modulating WRKY functions.

## 5 WRKYs regulate plant responses to abiotic stresses

Plants are routinely subjected to various stresses, which impair the normal growth and plant yield (Thomashow, 1999; Khoso et al., 2022). To surmount the stresses, sophisticated defense mechanisms have formed (Rushton et al., 2010; Schluttenhofer and Yuan, 2015; Sood et al., 2021). Recent research has shown that WRKYs have important functions in plant defenses against various stresses, including pathogens, cold, salinity, drought, and nutrition. The majority of WRKYs with known functions can be classified into three subfamilies, as shown in **Figure 1**; **Table 2**. Interestingly, WRKY members that regulate drought stress response are mostly found in subgroups 2b, 2c, 2d, and 2e, while those regulate salt resistance mainly belong to the 2a, 2c, and 3 subgroups. Additionally, WRKYs that regulate cold tolerance are mainly found in subgroups 1 and 2c, and those involved in plant responses to heavy metal poisoning are primarily found in subgroup 2c.

**Table 2:** WRKY involved in plant abiotic stresses response.

Species	Stress response	Nomenclature	Subgroup	Target gene	Regulation of target gene	Regulation of stress response	The effect of target gene	Function	References
*Arabidopsis thaliana*	Drought	MaWRKY80	2c	*NCEDs*	+	+	ABA-related gene	Participate in drought stress response	(Liu et al., 2020)
*Sorghum bicolor* (L.) Moench	Drought	SbWRKY30	3	*SbRD19*	+	+	Drought-related gene	Participate in drought stress response	(Yang et al., 2020)
*Arabidopsis thaliana*	Drought	WRKY46/54/70	3	*BR/BES1*	+	–	Drought-related gene	Participate in drought stress response	(Chen et al., 2017)
*Gossypium hirsutum*	Drought	GhWRKY21	2d	*GhHAB*	+	–	Drought-related gene	Participate in drought stress response	(Wang et al., 2021b)
*Oryza sativa*	Drought	OsWRKY11	2c	*RAB21*	+	+	Dehydrins gene	Participate in drought stress response	(Lee et al., 2018)
*Oryza sativa*	Drought	OsWRKY55	3	*OsAP2-39*	+	–	Drought-related gene	Participate in drought stress response	(Huang et al., 2021)
*Solanum lycopersicum* L.	Drought	SlWRKY81	3	*SlP5CS1*	–	–	Proline biosynthetic gene	Participate in drought stress response	(Ahammed et al., 2020a)
*Glycine max* (Linn.) Merr.	Drought	GmWRKY54	2c	*PYL8, SRK2A, CIPK11 and CPK3*	+	+	Help to integrate calcium signaling with ABA signaling	Participate in drought stress response	(Wei et al., 2019)
*Pyrus betulaefolia*	Drought	PbrWRKY53	3	*PbrNCED1*	+	+	ABA-responsive gene	Participate in drought stress response	(Liu et al., 2019b)
*Oryza sativa*	Drought	OsWRKY5	2b	*OsMYB2*	–	–	ABA-responsive gene	Participate in drought stress response	(Lim et al., 2022)
*Malus domestica* Borkh	Drought	MdWRKY17	1	*MdSUFB*	+	+	Inhibiting chlorophyll degradation and stabilizing electron transport during photosynthesis	Participate in drought stress response	(Shan et al., 2021)
*Iris germanica*	Drought	IgWRKY50	2c	*RD29A, DREB2A, PP2CA, and ABA2*	+	+	Stress-Related Gene	Participate in drought stress response	(Zhang et al., 2022c)
*Iris germanica*	Drought	IgWRKY32	1	*RD29A, DREB2A, PP2CA, and ABA2*	+	+	Stress-Related Gene	Participate in drought stress response	(Zhang et al., 2022c)
*Gossypium* spp	Drought	GhWRKY33	3	*ERD15 and SOS2*	–	–	Drought-related genes	Participate in drought stress response	(Wang et al., 2019)
*Phyllostachys edulis*	Drought	PheWRKY86	2c	*OsNCED1*	+	+	ABA-responsive gene	Participate in drought stress response	(Wu et al., 2022a)
*Gossypium hirsutum* L.	Drought	GhWRKY91	2e	*GhWRKY17*	+	+	Associate with ABA signals and reactive oxygen species production	Participate in drought stress response	(Gu et al., 2019)
*Oryza sativa*	Salt	OsWRKY54	3	*OsHKT1;5*	+	+	Participate in the Na^+^ transfer	Participate in salt stress response	(Huang et al., 2022a)
*Arabidopsis thaliana*	Salt	GarWRKY17	2c	*/*	+	+	/	Participate in salt stress response	(Fan et al., 2015)
*Arabidopsis thaliana*	Salt	GarWRKY104	2c	*/*	+	+	/	Participate in salt stress response	
*Populus alba* var. *pyramidalis*	Salt	PalWRKY77	2a	*PalNAC002, PalRD26*	–	–	ABA- and salt-related genes	Participate in salt stress response	(Jiang et al., 2021)
*Phyllostachys edulis*	Salt	PeWRKY83	2c	*/*	+	+	ABA-related genes	Participate in salt stress response	(Wu et al., 2017)
*Zea mays*	Salt	ZmWRKY20 and ZmWRKY115	3	*ZmbZIP111*	–	–	Salt-related genes	Participate in salt stress response	(Bo et al., 2022)
*Cucumis sativus* L.	Cold	CsWRKY46	2c	*ABI5/RD29A, COR47*	+	+	ABA-/cold-related genes	Participate in cold stress response	(Zhang et al., 2016)
*Cynodon dactylon*	Cold	CdWRKY2	2c	*CdSPS1, CdCBF1*	+	+	Mediate sucrose biosynthesis and CBF-signalling pathway	Participate in cold stress response	(Huang et al., 2022b)
*Oryza sativa*	Cold	OsWRKY63	1	*OsWRKY76*	–	–	Cold-related gene	Participate in cold stress response	(Zhang et al., 2022d)
*Brassica napus*	Boron	BnaA9.WRKY47	2b	*BnaA3.NIP5;1*	+	+	Facilitate efficient B uptake	Participate in boron stress response	(Feng et al., 2020)
*Capsicum annuum*	Phosphorus	CaWRKY58	1	*PHT1*	+	+	Phosphorus-deficiency related gene	Participate in phosphorus deficiency response	(Cai et al., 2021)
*Malus domestica*	Phosphorus	MdWRKY39	2b	*MdPHT1;7*	–	–	Phosphorus transporter-related gene	Participate in phosphorus deficiency response	(Zhou et al., 2023)
*Oryza sativa*	Phosphorus	OsWRKY21	3	*PHT1;1*	+	+	Facilitate phosphorus acquisition	Participate in phosphorus deficiency response	(Zhang et al., 2021a)
*Populus deltoides X Populus euramericana*	Phosphorus	PdeWRKY65	2e	*PdePHT1;9*	–	–	Pi transporter-related gene	Participate in phosphorus deficiency response	(Yang et al., 2022)
*Populus trichocarpa*	Phosphorus	PdeWRKY6	2b	*PdePHT1;9*	+	+	A positive regulator of Pi concentrations	Participate in phosphorus deficiency response	(Yang et al., 2022)
*Populus tomentosa Carr.*	Phosphorus	PtoWRKY40	2a	*PtoPHT1s*	–	–	Mediate Pi content increase	Participate in phosphorus deficiency response	(Chen et al., 2022)
*Oryza sativa*	Phosphorus	OsWRKY108	3	*PHT1;1*	+	+	Promote Pi accumulation	Participate in phosphorus deficiency response	(Zhang et al., 2021a)
*Arabidopsis thaliana*	Nitrogen	AtWRKY46	3	*GH3.1, GH3.6, UGT75D1, UGT84B2*	–	–	NUDX9 and IAA-conjugating genes	Participate in NH_4_^+^ tolerance response	(Di et al., 2021)
*Oryza sativa*	Phosphorus	OsWRKY28	2c	*/*	+	+	Phosphate-Responsive Genes	Participate in phosphorus deficiency response	(Wang et al., 2018)
*Arabidopsis thaliana*	Other	ABT1/WRKY14	2c	*/*	/	–	/	Participate in thermo morphogenesis response	(Qin et al., 2022)
*Arabidopsis thaliana*	Other	WRKY13	2c	*DCD*	+	+	Increase the production of H_2_S	Participate in cadmium stress response	(Zhang et al., 2020)

ABA, abscisic acid; ABI, abscisic acid-insensitive; AP2, APETALA-2-like transcription factor; BnaA3.NIP5;1, a Nodulin26-like intrinsic protein (NIP)-encoding gene as the responsible gene for B efficiency loci in B. napus cv. Qingyou10; BR/BES1, brassinosteroid-regulated transcription factor; bZIP, basic leucine zipper; CBF, C-repeat binding factor; CIPK, CBL-INTERACTING PROTEIN KINASE; COR, cold regulated; CPK, calcium-dependent kinase; DCD, D-CYSTEINE DESULFHYDRASE; DREB, dehydration-responsive element-binding; ERD, early responsive to dehydration; GH, glycoside hydrolase; HAB, clade-A-type PP2C (type 2C protein phosphatases); HKT, high-affinity K^+^ transporter; MYB, myb avian myeloblastosis viral oncogene homolog; NAC, No apical meristem (NAM), Arabidopsis transcription activation factor (ATAF), and Cup-shaped cotyledon (CUC); NCED, 9-cis-epoxycarotenoid dioxygenase; P5CS, pyrroline-5-carboxylate synthetase; PHT, phosphate transporter; PP2C, protein phosphatases 2C; PYL, pyrabactin resistance 1-like protein; RAB, RESPONSIVE TO ABA; RD, responsive to dehydration; SOS, salt overly sensitive; SPS, sucrose-phosphate synthase; SRK, S-locus receptor kinase; SUFB, sulfur mobilization (SUF) system that assembles Fe-S clusters; UGT, UDP-glucuronosyltransferase. The ‘+’ represents positive regulation, the ‘-’ represents negative regulation, and the ‘/’ represents unknown.

### 5.1 Involvement in drought stress

Drought stress results in cell dehydration, which threatens plant growth and yield worldwide. Long-term adaptation and evolution have led plants to have a variety of mechanisms to combat drought-induced water deficits, including closing stomata to curb water loss, a response mediated mostly by the abscisic acid (ABA) signaling (Lee et al., 1999; Finkelstein et al., 2002; Oztur et al., 2002; Shinozaki et al., 2003; Tang et al., 2016; Ahammed et al., 2020a). Under drought stress, the abscisic acid (ABA) signaling pathway often leads to stomatal closure (Ng et al., 2001; Qiu and Yu, 2009).

Actually, many WRKYs orchestrate plant drought responses by regulating ABA signaling. For instance, overexpressing of *GsWRKY20*, which was proven to improved response to ABA, could enhance plant tolerance to drought stress via stomatal closure (Luo et al., 2013). Besides, *TaWRKY146*-Overexpression also enhances drought tolerance through stomatal closure (Ma et al., 2017). In addition, soybean GmWRKY54 activates genes connected with ABA and Ca^2+^ pathways, alleviates water loss, and achieves drought resistance (Wei et al., 2019). PbrWRKY53 binds to and upregulates 9-cis-epoxycarotenoid dioxygenase1 (*PbrNCED1*), stimulating vitamin C biosynthesis and drought tolerance in Chinese white pear (*Pyrus communis*) (Liu et al., 2019b). In iris (*Iris germanica*), IgWRKY32 and IgWRKY50 together stimulate ABA signaling to upgrade drought tolerance (Zhang et al., 2022c). In contrast, the cotton GhWRKY21 and GhWRKY33, and the rice OsWRKY5, suppress this signaling, thereby undermining drought tolerance (Wang et al., 2019; Wang et al., 2021b; Lim et al., 2022).

Apart from stomatal and ABA regulation, a multitude of WRKYs can enhance plants tolerance to drought by regulating responsive genes in other pathways. Rice OsWRKY55 binds to and upregulates the APETALA-2-like transcription factor gene (*OsAP2-39)* to negatively modulate ethylene synthesis and drought tolerance (Huang et al., 2021). Sorghum (*Sorghum bicolor*) SbWRKY30 in induces the *Responsive to Dehydration 19* (*SbRD19*), a homolog of the Arabidopsis drought-responsive *RD19* (Yang et al., 2020). In apple, by modulating the iron-sulfur cluster biosynthesis protein gene (*MdSUFB)* expression, the MdMEK2-MdMPK6-MdWRKY17 cascade regulates chlorophyll levels during drought (Shan et al., 2021). Additionally in rice, OsWRKY11 directly upregulates RESPONSIVE TO ABA21 (*RAB21)*, enhancing drought tolerance (Lee et al., 2018). PheWRKY86 also upregulates *NCED1*, encoding a rate-limiting ABA biosynthetic enzyme, conferring drought tolerance (Wu et al., 2022a).

A number of WRKYs mediate drought responses by modulating osmolyte accumulation and ROS scavenging. For instance, SlWRKY81 and WRKY46/54/70 suppress proline biosynthesis and drought responses in tomato and Arabidopsis (Ahammed et al., 2020a). Conversely, in banana (*Musa acuminata*), MaWRKY80 upregulates ABA biosynthesis, osmolyte accumulation, and ROS detoxification, thereby enhancing drought tolerance (Chen et al., 2017; Liu et al., 2020).

In summary, these compelling findings highlight the significant roles of WRKYs, particularly in governing ABA signaling, osmolyte metabolism and ROS homeostasis, in calibrating plant adaptation to drought stress.

### 5.2 Regulation of plant resistance to salinity

The growing issue of soil salinization is having a negative impact on plant growth and reducing crop yields on a global scale. Salt stress can significantly hinder plant growth, leading to harmful consequences for agricultural production worldwide (Hasegawa et al., 2000; Yan et al., 2022).

Several WRKYs were revealed as positive regulators in plant tolerance to salinity. Employing CRISPR-Cas9 to knockout *OsWRKY54*, Huang et al. (2022a) revealed its beneficial effect on conferring rice tolerance to salinity. Similarly, by upregulating *GarWRKY17* and *GarWRKY104*, the salt tolerance of Arabidopsis was enhanced at different developmental stages (Fan et al., 2015). Wu et al. (2017) found that overexpressing *PeWRKY83* in Arabidopsis substantiated salt tolerance, resulting in increased proline accumulation, higher germination rates, less electrolyte leakage, and lower membrane damage under salt stress.

In contrast, WRKYs could also act as negative regulators on salt resistance. Jiang et al. (2021) demonstrated that overexpressing of *PalWRKY77* in poplar compromised salt tolerance through inhibition of ABA-responsive genes. Bo et al. (2022) indicated that the maize (*Zea mays*) ZmWRKY20-ZmWRKY115 complex in nucleus bound to promoters of basic leucine zipper (*ZmbZIP111*) to inhibit the expression of *ZmbZIP111*, which elevated the salt sensitivity of maize seedlings.

### 5.3 Orchestrating plant responses to cold stress

Cold stress is unfavorable to normal plant development and poses a major constraint on agricultural productivity (Andaya and Mackill, 2003). Plants have evolved various physiological, biochemical and molecular adaptation mechanisms to improve cold tolerance (Ishitani et al., 1997; Ding et al., 2019; Ding and Yang, 2022; Khoso et al., 2022). Analyzing the regulatory mechanisms and elucidating the transcriptional networks has uncovered many cold stress-related genes (Ritonga et al., 2021).

In particular, WRKYs are instrumental to cold tolerance across plant species. WRKY members in 1 and 2c, were found to regulate the chilling stress. Overexpressing *CsWRKY46* from cucumber (*Cucumis sativus* L.) caused higher tolerance to freezing by upregulating the expression of Responsive to Desiccation gene (*RD29A)* and cold regulated 47 gene (*COR47)*, and by positively regulating expression of some ABA-regulated genes under low temperature stress (Zhang et al., 2016). By contrast, rice OsWRKY63 downregulated various genes related to chilling response and ROS-scavenging, and negatively regulated chilling tolerance via the WRKY63-WRKY76-DREB1B regulatory cascade (Zhang et al., 2022d). CdWRKY2 positively regulated cold responses by binding to promoters of sucrose phosphate synthase1 (*CdSPS1)* and C-repeat binding factor 1 (*CdCBF1)* in bermudagrass *(Cynodon dactylon)*, thereby coordinating sucrose biosynthesis and the CBF pathway (Huang et al., 2022b).

### 5.4 Fine-tuning plant responses to nutrient deficiency and other stresses

Insufficient or excessive levels of soil nutrients impede plant growth. Nutrient deficiency symptoms in plants vary from element to element. As one of the important plant nutrients, phosphorus deficiency affects the photosynthetic rate of plant leaves, the growth of plant stems and the formation of reproductive organs(Barry, 1988; Lauer et al., 1989). Besides, deficiencies of boron or nitrogen (NH_4_^+^) also seriously affect plant growth (Tanada, 1978; Krueger et al., 1987; Zhang et al., 2021b).

WRKY also has multiple roles in regulating these plant nutrients. For instance, suppression of *OsWRKY28* resulted in decreased phosphate (Pi) accumulation in rice (Wang et al., 2018). In addition, OsWRKY21 and OsWRKY108 can positively regulate the expression level of phosphate transporter (*OsPHT1;1*) for Pi accumulation (Zhang et al., 2021a). In apple, overexpression of *MdWRKY39* led to phosphorus deficiency through up-regulating *MdPHT1;7* (Zhou et al., 2023). In poplar, a phosphate starvation response 1 (PHR1) homolog can interact with PtoWRKY40 to inhibit the transcription of *PtoPHT1*, thereby mediating an increase in Pi content (Chen et al., 2022). Besides, PdeWRKY6 and PdeWRKY65 modulated tissue Pi concentration by coordinating the expression of *PdePHT1;9* (Yang et al., 2022). CaWRKY58 activated *PHT1* and coordinated with Ca14-3-3 to improve Pi concentration under the Pi-insufficient conditions (Cai et al., 2021). In addition, BnaA9.WRKY47 enhanced the tolerance of rapeseed to boron deficiency by upregulating the boric acid channel gene (*BnaA3.NIP5;1)*, thus increasing boron uptake (Feng et al., 2020). AtWRKY46 suppressed the expression of GDP-D-mannose pyrophosphohydrolase (*NUDX9)* and IAA-conjugating genes, resulting in root tolerance to NH_4_^+^ (Di et al., 2021).

WRKY also play important roles in regulating plant responses to other stresses, including heat and cadmium toxicity. Qin et al. (2022) hypothesized that Arabidopsis ABT1/WRKY14 plays a key negative regulatory role in plant thermomorphogenesis. Zhang et al. (2020) demonstrated that Cd induces WRKY13 to activate *D-CYSTEINE DESULFHYDRASE (DCD)* expression to elevate H_2_S level and enhance Cd tolerance in Arabidopsis.

## 6 Mechanism of WRKY affecting flavonoid synthesis

Plants are inevitably challenged by various environmental stresses including drought, high salinity, cold, ultraviolet (UV) radiation damage (Apel and Hirt, 2004; Torres and Dangl, 2005; Ferreyra et al., 2012; Choudhury et al., 2017; Song et al., 2022; Sugimoto et al., 2022). Recent evidence indicates that flavonoids, a class of antioxidants, are capable of scavenging the overproduced ROS and alleviating oxidative injury (Pi et al., 2016; Pi et al., 2018; Pi et al., 2019; Yu et al., 2020; Qian et al., 2021; Wu et al., 2022b).

### 6.1 Regulation of flavonoid biosynthesis in plants

**Figure 3:**
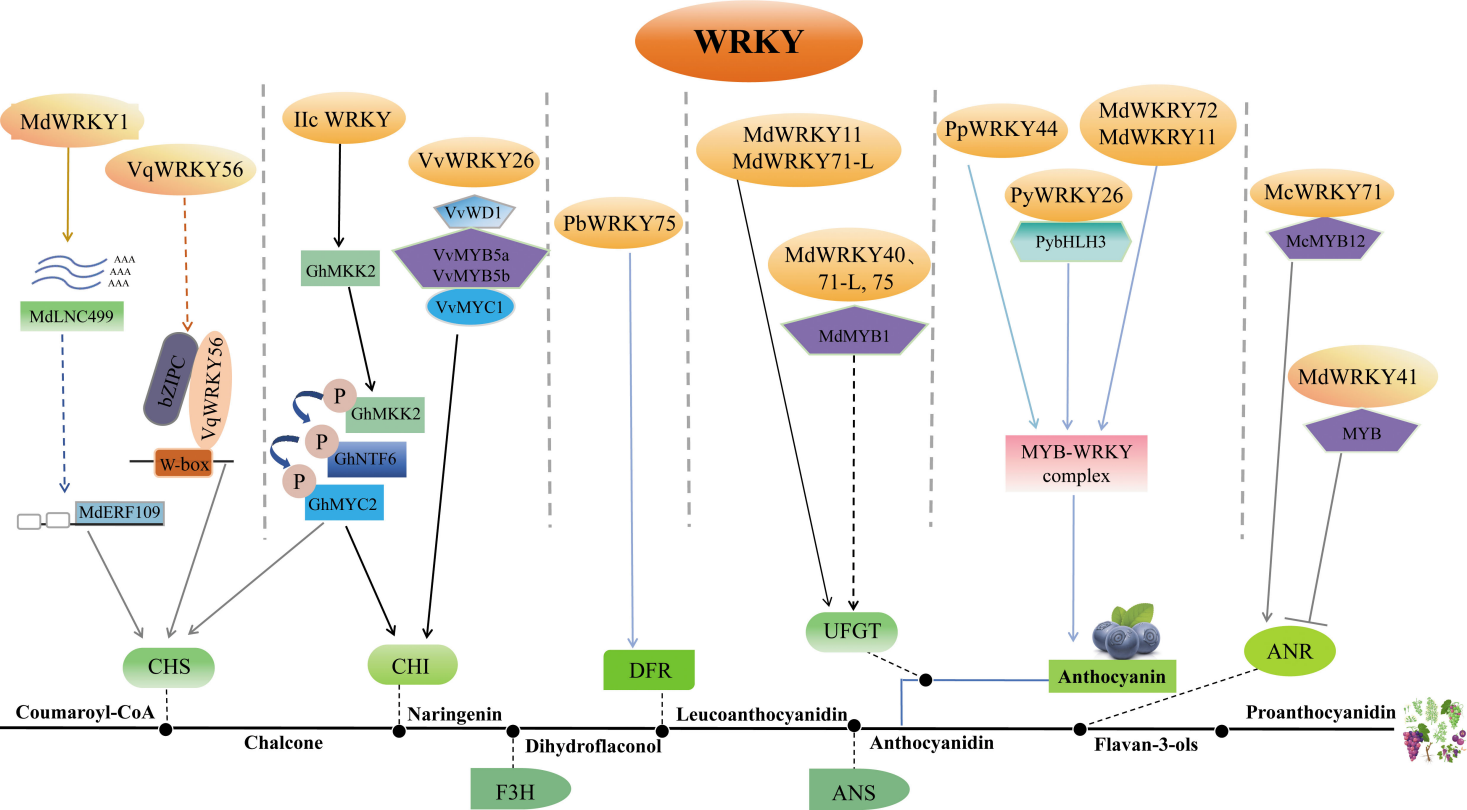
Depiction of the role of WRKY in the biosynthesis of flavonoid. The complex role of WRKY in regulating flavonoid biosynthesis highlights its crucial function in this pathway and subsequent stress responses in plants. The WRKY protein modulates flavonoid biosynthesis by controlling the expression of flavonoid biosynthesis genes in various ways. MdWRKY1 and VqWRKY56 can influence the transcription of *CHS*, while VvWRKY26 can control *CHI* expression by forming the MBWW complex. PbWRKY75 might enhance the expression of *DFR*. MdWRKY11, MdWRKY71-L, MdWRKY40, and MdWRKY75 can directly or indirectly control the expression of *UFGT*. MdWRKY72, MdWRKY11, PpWRKY44, and PyWRKY26 can impact anthocyanin synthesis by forming the MYB-WRKY complex. In addition to positive regulation, MdWRKY41 also has a role in negatively regulating ANR synthesis. All these pathways influence the synthesis of anthocyanin or proanthocyanidin ultimately. Arrows represent positive regulation, and T-bars represent negative regulation. CHS, chalcone synthase; CHI, chalcone isomerase; F3H: flavanone 3-hydroxylase; DFR, dihydroflavonol 4-reductase; ANS, anthocyanidin synthase; ANR, anthocyanin reductase; UFGT, flavonoid 3-O-glycosyl-transferase.

Flavonoids encompass several subclasses, including anthocyanins, proanthocyanidins, flavones, flavanols, flavonols, flavanones and isoflavones (Panche et al., 2016; Durazzo et al., 2019; Luo et al., 2019). More than 10, 000 diverse variants are generated by glycosylation and other modifications (Weston and Mathesius, 2013). Anthocyanins in particular confer bright colors to plant tissues and also act as antioxidants. Flavonoid biosynthesis proceeds through a branched pathway catalyzed by sequential enzymatic reactions. The biosynthesis of flavonoid is regulated by an intricate transcriptional network. Distinct WRKYs can integrate in various ways and exert different regulatory effects on flavonoid synthesis (**Table 3**; **Figure 3**).

**Table 3:** Mechanism of WRKY on metabolism of flavonoids.

Species	WRKY name	Subgroup	Regulation of target gene	Regulation of biological	Target gene	Function	References
*Arabidopsis thaliana*	AtWRKY41	3	/	–	/	Negative regulation of anthocyanin biosynthesis	(Duan et al., 2018)
*Malus crabapple*	McWRKY71	2c	+	+	*McANR*	Enhance proanthocyanidin biosynthesis	(Zhang et al., 2022b)
*Malus domestica*	MdWRKY75	2c	+	+	*MdMYB1*	Enhance anthocyanin accumulation	(Su et al., 2022b)
*Malus domestica*	MdWRKY71-L	2c	+	+	*MdUFGT* and *MdMYB1*	Enhance anthocyanin accumulation	(Su et al., 2022a)
*Malus domestica*	MdWRKY40	2a	+	+	*MdUFGT*	Promote wounding-induced anthocyanin biosynthesis	(An et al., 2019)
*Malus domestica*	MdWRKY11	2d	+	+	*MdUFGT*	Enhance anthocyanin accumulation	(Liu et al., 2019a)
*Malus domestica*	MdWRKY41	3	–	–	*MdANR, MdUFGT* and *MdMYB12*	Negatively regulates anthocyanin and PA biosynthesis	(Mao et al., 2021)
*Malus domestica*	MdWRKY72	2b	+	+	*MdHY5* and *MdMYB1*	Enhance anthocyanin accumulation	(Hu et al., 2020)
*Malus domestica*	MdWRKY1	2d	/	+	/	Enhance anthocyanin accumulation	(Ma et al., 2021a)
*Vitis vinifera*	VvWRKY26	1	+	+	*VvCHI*	Improved activation efficiency and flavonoid accumulation	(Amato et al., 2019)
*Vitis vinifera*	VqWRKY56	2b	+	+	*VvCHS3, VvLAR1*, and *VvANR*	Promote proanthocyanidin biosynthesis and increase resistance to powdery mildew	(Wang et al., 2023d)
*Pyrus L.*	PpWRKY44	1	+	+	*PpMYB10*	Stimulating anthocyanins	(Alabd et al., 2022)
*Pyrus bretschneideri*	PbWRKY75	2c	+	+	*PbDFR, PbUFGT*, and *PbMYB10b*	Promote anthocyanin synthesis	(Cong et al., 2021)
*Pyrus L.*	PyWRKY26	1	+	+	*PyMYB114*	Promote anthocyanin synthesis	(Li et al., 2020a)

ANR, anthocyanin reductase; ANS, anthocyanidin synthase; CHI, chalcone isomerase; CHS, chalcone synthase; DFR, dihydroflavonol 4-reductase; F3H, flavanone 3-hydroxylase; HY, ELONGATED HYPOCOTYL; LAR, leucine anthocyanin reductase; MYB, myb avian myeloblastosis viral oncogene homolog; UFGT, flavonoid 3-O-glycosyl-transferase. The ‘+’ represents positive regulation, the ‘-’ represents negative regulation, and the ‘/’ represents unknown.

WRKYs directly regulate flavonoid synthesis by transactivation of related enzyme genes. In apple, MdWRKY11 regulates anthocyanin synthesis through directly binding to the flavonoid 3-O-glycosyl-transferase (*UFGT)* promoter (Liu et al., 2019a); MdWRKY40 binds *anthocyanidin synthase* (*MdANS*) promoter (Zhang et al., 2019); and MdWRKY41 negatively regulates anthocyanin and proanthocyanidins biosynthesis by binding W-boxes of apple *anthocyanin reductase (MdANR*), *MdUFGT*, myb avian myeloblastosis viral oncogene homolog 12 (*MdMYB12)* (Mao et al., 2021); MdWRKY71-L targets *MdUFGT* (Su et al., 2022a). In crabapple (*Malus crabapple*), McWRKY71 controls McANR and proanthocyanidin synthesis (Zhang et al., 2022b). In grape, VqWRKY56 binds *chalcone synthase 3* (*VvCHS3*), *leucine anthocyanin reductase1* (*VvLAR1*) and *VvANR*, inducing proanthocyanidins (Wang et al., 2023d). In pear, PyMYB10 activates pear anthocyanin structural gene (Feng et al., 2010). PpWRKY44 activates *PpMYB10* by binding to its promoter for light-induced anthocyanin accumulation (Alabd et al., 2022). PbWRKY75 has shown to promote anthocyanin synthesis in *pear* by binding the promoters of *dihydroflavonol 4-reductase* (*PbDFR*) and *PbUFGT* (Cong et al., 2021). FaWRKY71 stimulates anthocyanin accumulation in strawberry (*Fragaria×ananassa*) by upregulating genes in the synthetic pathway [*flavonoid 3’-hydroxylase* (*FaF3’H*), *FaLAR*, *FaANR*, anthocyanin transporter genes *transparent testa* 19 (*FaTT19*) and *transparent testa* 12 (*FaTT12*)] (Yue et al., 2022).

WRKYs also influence flavonoid biosynthesis indirectly through modulation of other regulators. In apple, MdWRKY1 activates a long noncoding RNA (*MdLNC499)* and *MDERF109* expression, which in turn increases anthocyanin accumulation by inducing the expression of *MdUFGT*, *MdCHS* and basic helix-loop-helix 3 (*MdbHLH3)* (Ma et al., 2021a); MdWRKY71-L regulates anthocyanin synthesis via the ELONGATED HYPOCOTYL 5 (MdHY5)-MdMYB1 cascade; MdWRKY40 forms homodimers that bound two W-boxes in *MdANS* promoters, mitigating MdMYB111 inhibition of *MdANS* (Zhang et al., 2019); MdWRKY75 stimulates anthocyanins by associating with the *MdMYB1* promoter (Su et al., 2022a); MdWRKY72 binds *MdHY5* and *MdMYB1* (Hu et al., 2020); and MdWRKY11 binds with *MdHY5* (Liu et al., 2019a). In pear, PpWRKY44 upregulates *PpMYB10* to stimulate anthocyanins (Alabd et al., 2022); PyWRKY26 in conjunction with PybHLH3 targets the *PyMYB114* promoter, thus affecting anthocyanins accumulation (Li et al., 2020a). In Arabidopsis, the *WRKY41* mutation heightens anthocyanin levels, indicating AtWRKY41 represses anthocyanin synthesis by regulating *AtMYB75*, *AtMYB111*, *AtMYBD*, *AT1G68440* and *AtGSTF12* (Duan et al., 2018). In cotton, Group IIc WRKYs induce flavonoids by controlling protein kinase kinase 2 (*GhMKK2)*, a signaling kinase (Wang et al., 2022). In crabapple, McWRKY71 regulates proanthocyanidin synthesis by interacting with McMYB12 (Zhang et al., 2022b).

Furthermore, WRKY forms transcriptional complexes with other transcription factors. MYB, bHLH and WD40 compose the MBW complex to modulate PA synthesis and anthocyanin (Nesi et al., 2001; Ramsay and Glover, 2005; Gou et al., 2011). WRKYs participate in regulating the MBW complex. For example, VvWRKY26 is absorbed into the MBWW complex by VvMYB5a to regulate flavonoid hydroxylation in grape (Amato et al., 2017; Amato et al., 2019).

Thus, the biosynthesis of flavonoids is regulated by a complex transcriptional network. WRKYs utilize various strategies, such as directly binding to target promoters, interacting with other regulators, or forming transcriptional complexes, to regulate flavonoid biosynthesis. The combined efforts of multiple WRKYs and the interactions with other transcription factors allow for precise control over this metabolic pathway.

### 6.2 Elevation of plant stress tolerances through flavonoid synthesis

WRKYs play pivotal roles in modulating the synthesis of various flavonoid for regulating plant responses to various abiotic stresses caused by UV-B, O_3_, and wounding, etc. UV-B-induced apple MdWRKY71-L promotes in apple anthocyanin accumulation by directly activating *MdUFGT* and *MdMYB1* (Su et al., 2022a). When exposed to UV-B radiation in the transgenic calli, MdWRKY72 directly control anthocyanin synthesis via promoting MdMYB1, or indirectly regulates by interacting with MdMYB16 (Hu et al., 2020; Mao et al., 2021). In addition, McWRKY71 directly binds to the *McANR*, thus regulate the PA biosynthesis in regard to O_3_ stress in crabapple (Zhang et al., 2022b). Moreover, MdWRKY40, interacting with MdMYB1, enhance the activation of target genes in reaction to injuries (An et al., 2019).

Apart from abiotic stresses, WRKY also partakes in biotic stresses by regulating the biosynthesis of flavonoids. For example, expression of *VqWRKY56* activates PA biosynthesis genes (*VvCHS3*, *VvLAR1* and *VvANR*) (Wang et al., 2023d). The upregulation of GhMYC2 by group IIc WRKYs induced GhMKK2-GhNTF6 signaling and increased cotton resistance to Fusarium oxysporum via enhancing flavonoid biosynthesis. This demonstrates a novel defense mechanism mediated by WRKY-MAPK-regulated flavonoid biosynthesis against pathogen infection in cotton (Wang et al., 2022).

In short, WRKYs participate in almost all stages of flavonoid synthesis and regulate flavonoid synthesis genes through diverse mechanisms. However, the contribution of WRKY-mediated flavonoid synthesis to plant tolerance has been poorly characterized.

## 7 Conclusion and perspective

In this review, we optimized existing WRKY phylogenetic trees and tried to deduce unifying themes of distinct WRKY subfamilies governing specific stress responses and flavonoid metabolism.

Analysis of documented data reveals WRKY members across all subgroups participate in flavonoid synthesis. However, WRKYs regulating salt tolerance mainly belong to subgroups 2a, 2c and 3. Given their shared protein motifs (**Tables 2**, **3**; **Figure 1**), it is reasonable to hypothesize these WRKY subgroups promote flavonoid accumulation to enhance plant salinity tolerance. Similar hypotheses could be proposed for WRKY homologs tuning flavonoids to elevate plant resistance to cold, drought and nutrient deficiency. However, only a handful of studies have demonstrated direct relationships between WRKY-modulated flavonoid synthesis and stress tolerance. Perhaps the significant contribution of flavonoids has been overlooked in analyzing stress resistance, and roles of the WRKY-flavonoid interplay in plant stress tolerance deserve greater attention in future work. Nonetheless, laboratory validation remains necessary to verify these hypotheses.

On the other hand, research on WRKY-mediated flavonoid regulation has focused on few species like apple, Arabidopsis, grapevine and crabapple. Could WRKY regulate flavonoid synthesis in other species via distinct pathways? Molecular mechanisms underlying flavonoid-enhanced plant stress resilience remain largely unclear. Particularly, flavonoid regulation by WRKY transcription factors and subsequent impacts on plant stress response warrant deeper exploration.

Further research is imperative to elucidate the complex crosstalk between flavonoid metabolism and stress signaling cascades. In subsequent studies, integrating transcriptomic and metabolomic analyses could prove insightful. Transcriptomics can provide comprehensive information about WRKY transcription factors and identify flavonoid pathway target genes. Metabolomics can directly assess functional outcomes of WRKY-mediated flavonoid regulation. Together, these efforts will uncover valuable knowledge for engineering flavonoid pathways to improve multiple stress tolerance in economically important crops.

## Author contributions

JZ: Writing – original draft. HZ: Writing – original draft. LC: Writing – original draft. JL: Writing – review & editing. ZW: Writing – original draft. JP: Writing – original draft. FY: Writing – original draft. XN: Writing – review & editing. YW: Writing – review & editing. YHW: Writing – review & editing. RL: Writing – review & editing. EP: Funding acquisition, Resources, Writing – original draft. SW: Funding acquisition, Writing – original draft.
